# The conundrum of Identitive Dissociative Disorder: about a case

**DOI:** 10.1192/j.eurpsy.2022.2255

**Published:** 2022-09-01

**Authors:** P. Albarracin, E. Herrero Pellón, R. Galerón, M. Huete Naval, B. Serván

**Affiliations:** 1 Hospital Universitario Clínico San Carlos, Psychiatry And Mental Health, Madrid, Spain; 2 Hospital Clínico San Carlos, Institute Of Psychiatry And Mental Health, Madrid, Spain

**Keywords:** Trauma, identity dissociative disorder, dissociative symptoms, Psychosis

## Abstract

**Introduction:**

We present the case of a 22 year old male with a history of two hospitalizations in the Psychiatric ward of our hospital with psychotic symptoms that led to a diagnosis of schizophrenia, whose later evolution arose doubts about such a diagnosis and provoked a re-examination of the case, eventually leading to a diagnosis of Dissociative Identity Disorder.

**Objectives:**

To present a complex case of Identity Dissociative Disorder disguised by a myriad of psychotic-like symptom and to review the links between this kind of disorders and a personal history of trauma.

**Methods:**

We performed an extensive review of the scientific literature available regarding the topic of Dissociative Identity Disorder, using sources both in English and Spanish languages.

**Results:**

Our patient experimented two admissions into our Psychiatric ward due to acute psychopathological symptoms (auditive pseudohallucinations and visual hallucinations attributed by the patient to two different people who could influence on his behaviour), then linked to a début of a Paranoid Schizophrenia. The follow-up of the patient in a Day Clinic related to our hospital revealed a close relationship between the described symptoms and a personal history of trauma, as well as a lack of effect of the antipsychotic medication prescribed, and the clinical case eventually evolved to the development of two distinct identities within our patient, leading to a new working diagnose of Identity Dissociative Disorder.

**Conclusions:**

Identitive Dissociative Disorder is a complex, underestimated entity of difficult diagnosis with deep roots in personal traumatic history and whose multifaceted presentation may entail a challenge to clinicians.

**Disclosure:**

No significant relationships.

